# Chemoresponse after non-curative gastrectomy for M1 gastric cancer

**DOI:** 10.1186/s12957-015-0447-3

**Published:** 2015-01-30

**Authors:** Hyun Beak Shin, Seung Hyoung Lee, Young Gil Son, Seung Wan Ryu, Soo Sang Sohn

**Affiliations:** Department of Surgery, Keimyung University School of Medicine, 194, Dongsan-dong, Choong-gu, Daegu 700-712 Korea

**Keywords:** Chemoresponse, Non-curative gastrectomy, M1 gastric cancer, Chemotherapy, Survival

## Abstract

**Background:**

M1 gastric cancer has a poor oncologic outcome with a median survival of less than 1 year despite aggressive chemotherapy. Recent trials include chemotherapy combined non-curative gastrectomy. This study evaluated the chemoresponse after non-curative gastrectomy in M1 gastric cancer and the survival benefit.

**Methods:**

Between January 2000 and December 2010, 660 patients received chemotherapy for gastric cancer at the Department of Hemato-Oncology, Dongsan Medical Center, Keimyung University School of Medicine, Daegu, Korea. Data was collected retrospectively from the medical records. Patients who received preoperative or adjuvant chemotherapy, who underwent other surgeries like gastrojejunal bypass or exploratory laparotomy, who died within 3 months due to seriously advanced gastric cancer, who were lost to follow-up, or whose medical records were unsuitable for data collection were excluded. The remaining 101 patients had received chemotherapy only (CTx group, *n* = 76) or chemotherapy after non-curative gastrectomy (NCG + CTx group, *n* = 25). Clinicopathologic characteristics, chemoresponse, and overall survival were compared between the two groups.

**Results:**

There were no significant differences between the two groups in clinicopathologic characteristics including age, sex, body mass index (BMI), comorbidity, histologic differentiation, tumor location, clinical T stage, and initial site of distant metastasis. Chemoresponse was checked on two separate occasions from the initiation of chemotherapy: first chemotherapy regimen and until the third regimen change. The NCG + CTx group showed more favorable chemoresponse than the CTx group in both checks (60% and 72% vs. 18.4% and 23.7%). The NCG + CTx group showed longer overall survival than the CTx group (26 vs. 11 months).

**Conclusions:**

Non-curative gastrectomy in M1 gastric cancer could improve chemoresponse and extend overall survival.

## Background

M1 gastric cancer is characterized by distant metastasis at sites other than regional lymph node (LN) [1]. Distant metastasis comprises peritoneal metastasis including ovarian metastasis; hematogenous metastasis that spreads to the liver, lung, and bone; and metastasis to distant LNs including paraaortic, neck, and mediastinal LNs.

Chemotherapy has been recommended as a main treatment modality for M1 gastric cancer in the third Japanese gastric cancer treatment guidelines as well as in the 2013 National Comprehensive Cancer Network (NCCN) gastric cancer guidelines [2,3]. However, the median survival of M1 gastric cancer is under 1 year despite aggressive chemotherapy. Although a few clinical trials showed the effectiveness of adjuvant chemotherapy after curative resection in advanced gastric cancer [4-7], the generally poor oncologic outcomes in M1 gastric cancer might be caused by the lack of an outstanding chemotherapeutic agent and definite treatment guidelines specifying surgery [8].

Several study groups have tried to improve the oncologic outcomes with the various new concepts including liver resection for hepatic metastasis from gastric cancer, aggressive surgery with peritonectomy for localized peritoneal metastasis, intraperitoneal chemotherapy, surgery with curative intent in patients who have presented favorable response after chemotherapy, and chemotherapy after non-curative gastrectomy. Some survival benefits were reported [9-24]. But these studies focused on patient survival, although chemoresponse could be the most important mechanism to prolong the survival time after chemotherapy.

With this in mind, we concentrated our attention on the chemoresponse after non-curative gastrectomy in M1 gastric cancer. We hypothesized that non-curative gastrectomy will improve the chemoresponse by reducing tumor burden, similar to other cancers, with non-curative gastrectomy inhibiting the chemoresponse by destroying the lymphatic channels or blood vessels that are the anatomic routes to tumor. Accordingly, the goal was to identify if non-curative gastrectomy could improve the chemoresponse. The influence of chemoresponse on patient survival was assessed.

## Methods

**Figure 1:**
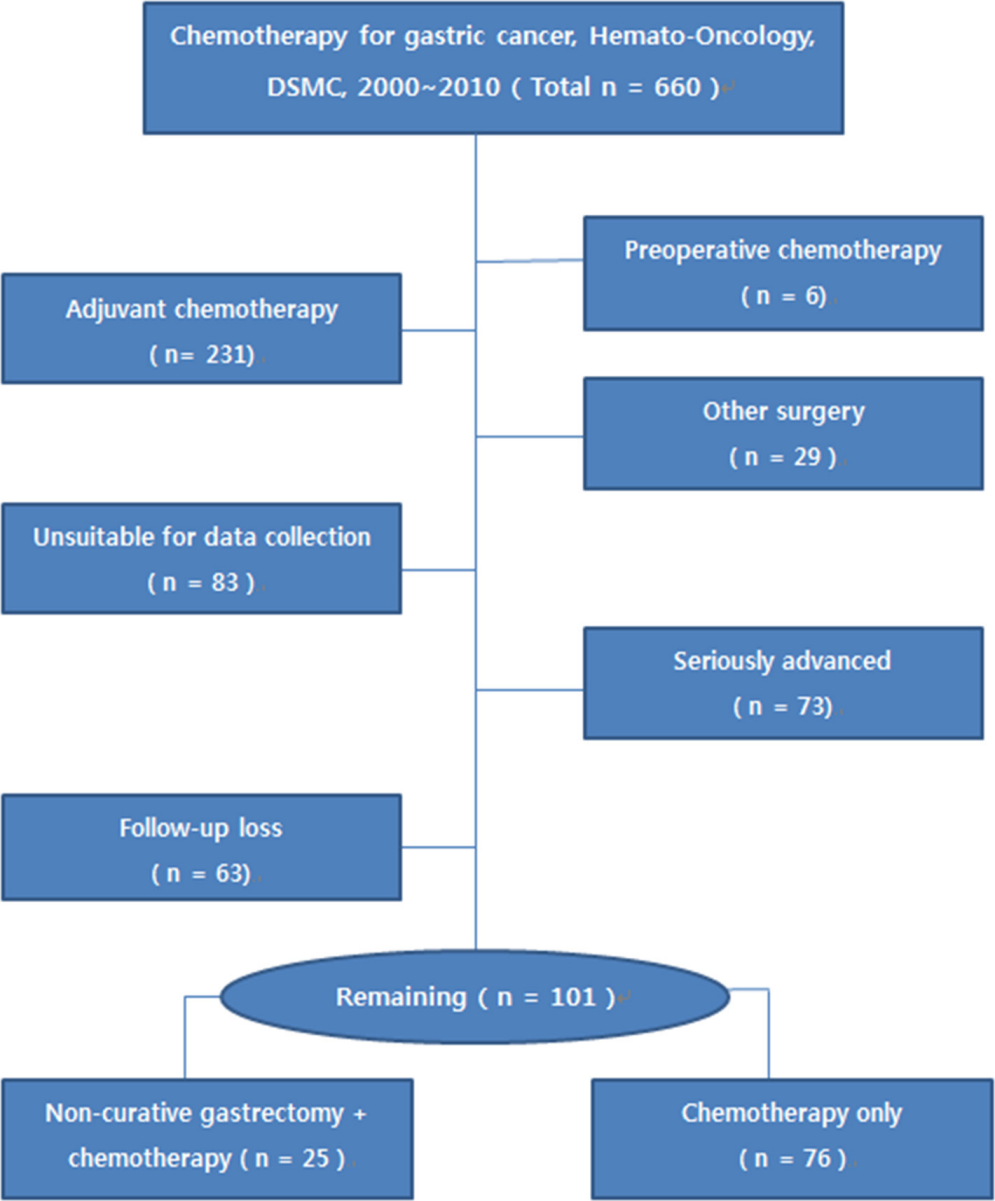
**Study design.**

Between January 2000 and December 2010, 660 patients received chemotherapy for gastric cancer at the Department of Hemato-Oncology, Dongsan Medical Center, Keimyung University School of Medicine, Daegu, Korea. We collected the data from this group through a retrospective review of medical records. Patients who received preoperative or adjuvant chemotherapy, who underwent other surgeries like gastrojejunal bypass or exploratory laparotomy, who died within 3 months due to seriously advanced gastric cancer, and who were lost to follow-up, whose medical records were unsuitable for data collection were excluded (Figure 1). The remaining 101 patients had received chemotherapy only (CTx group, *n* = 76) or chemotherapy after non-curative gastrectomy (NCG + CTx group, *n* = 25). Non-curative gastrectomies were comprised of total gastrectomy (*n* = 15) and subtotal gastrectomy (*n* = 10).

**Figure 2:**
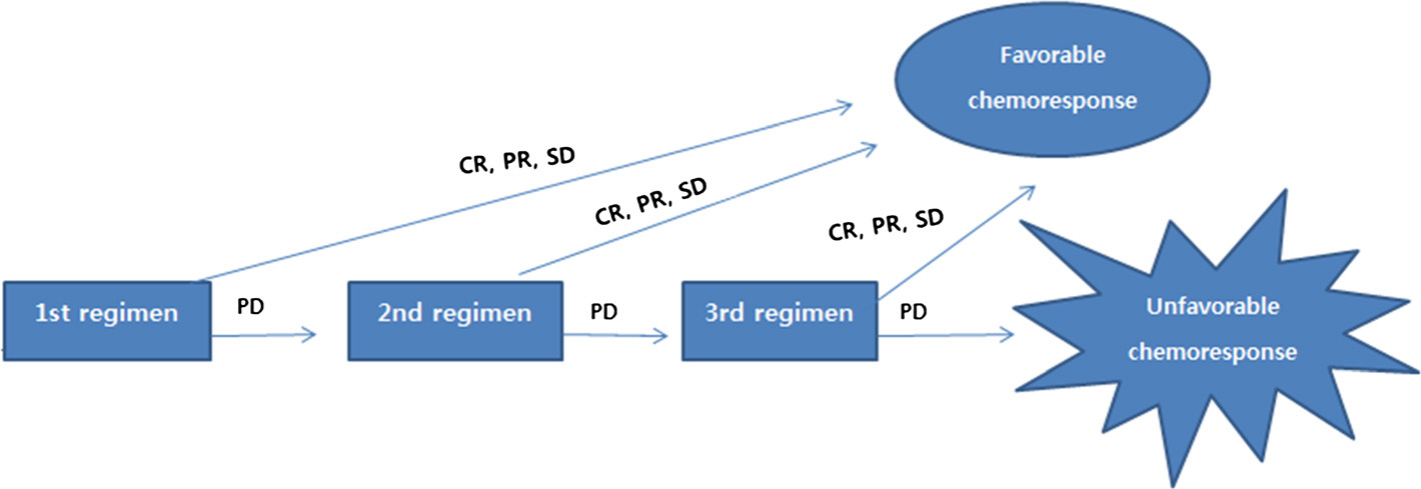
**Reclassification of chemoresponse according to the Response Evaluation Criteria in Solid Tumor (RECIST).**

There were no significant differences between the two groups in clinicopathologic characteristics including age, sex, body mass index (BMI), comorbidity, histologic differentiation, tumor location, clinical T stage, and initial site of distant metastasis (Table 1). Chemoresponse and overall survival were compared. In the absence of previously defined criteria for chemoresponse, new criteria were assigned (Figure 2). Patients presenting once with complete response (CR) or partial response (PR) or stable disease (SD) on the Response Evaluation Criteria in Solid Tumors (RECIST) according to clinical progress and follow-up computed tomography (CT), positron emission tomography (PET), bone scan, or magnetic resonance imaging (MRI) from initiation of chemotherapy to the time of evaluation were reclassified as ‘favorable chemoresponse.’ Otherwise, patients who continued to present progressive disease (PD) until evaluation were reclassified as ‘unfavorable chemoresponse’. The clinical course of patients who received palliative chemotherapy for M1 gastric cancer is typically determined following the third change of regimen. Thus, chemoresponse was checked on two separate occasions from the initiation of chemotherapy. One was chemoresponse for the first chemotherapy regimen. The other was chemoresponse until the third regimen change. Overall survival between the two groups was compared. Various regimens were applied for the first chemotherapy as shown in Table 2.

**Table 1:** **Clinicopathologic characteristics**

		**CTx (** ***n*** **= 76)**	**NCG + CTx (** ***n*** **= 25)**	***P*** **value**
Age, years	Mean (SD)	53.7 (±10.9)	54.3 (±11.0)	>0.05
Sex	Male	54 (71.1%)	18 (72.0%)	>0.05
	Female	22 (28.9%)	7 (28.0%)	
BMI, kg/m^2^	Mean (SD)	22.0 (±3.2)	21.8 (±2.2)	>0.05
Comorbidity	Yes	14 (18.4%)	6 (24.0%)	>0.05
Histology	Differentiated	17 (22.4%)	7 (28.0%)	>0.05
	Undifferentiated	59 (77.6%)	18 (72.0%)	
Tumor location	Upper	11 (14.5%)	7 (28.0%)	>0.05
	Middle	13 (17.1%)	1 (4.0%)	
	Lower	39 (51.3%)	13 (52.0%)	
	Entire	13 (17.1%)	4 (16.0%)	
cT stage	T1 ~ 3	7 (9.2%)	7 (28.0%)	<0.05
	T4	69 (90.8%)	18 (72.0%)	
Initial M1 site	Peritoneal	19 (25.0%)	15 (60.0%)	<0.01
	Hematogenous	15 (19.7%)	5 (20.0%)	
	Distant LN	24 (31.6%)	2 (8.0%)	
	Mixed	18 (23.7%)	3 (12.0%)	

*Abbreviation*: *cT stage* clinical T stage, *SD* standard deviation.

**Table 2:** **Regimens used for the first chemotherapy**

**Regimen**	**Total (** ***n*** **= 101)**	**CTx (** ***n*** **= 76)**	**NCG + CTx (** ***n*** **= 25)**
Paclitaxel/cisplatin/TS-1	27	24	3
Paclitaxel/cisplatin	21	21	
Capecitabine/cisplatin	9	2	7
FOLFOX (folinic acid/5-FU/oxaliplatin)	7	2	5
Paclitaxel/TS-1	6	5	1
Irinotecan/cisplatin	5	4	1
Docetaxel/oxaliplatin	5	4	1
5-FU/cisplatin	4	3	1
Heptaplatin/5-FU	4	4	
FOLFIRI (folinic acid/5-FU/irinotecan)	3	3	
Docetaxel/5-FU/cisplatin	2	2	
TS-1	2		2
FOLFOX/cetuximab	1	1	
Capecitabine/lapatinib/eloxatin	1		1
MFC (MMC/5-FU/cytarabine)	1		1
Docetaxel/cisplatin	1		1
TS-1/cisplatin	1		1
Capecitabine/lapatinib	1	1	

*Abbreviations*: *5-FU* 5-fluorouracil, *MMC* mitomycin-C, *TS-1* tegafur-gimeracil-oteracil potassium.

This study was approved by the Institutional Review Board of Keimyung University School of Medicine, Dongsan Medical Center, Daegu, Korea (IRB file no. 2014-01-018).

### Statistical analyses

SPSS version 20.0 (SPSS, Chicago, IL, USA) was used for statistical analyses. To compare the clinicopathologic characteristics and the chemoresponse between the two groups, chi-square test was used for categorical variables, and Student’s *t*-test and Fisher’s exact test were used for continuous variables. Overall survival was analyzed with the Kaplan-Meier curve analysis, and statistical significance was evaluated with log-rank test. *P* < 0.05 was considered significant.

## Results

There were no significant differences in age, sex, BMI, comorbidity, histologic differentiation, and tumor location between the two groups (Table 1). Despite efforts to make the two groups homogenous, clinical T4 gastric cancer was somewhat more prevalent in the CTx group. However, considering that serosal exposure of gastric cancer could not be identified directly in this group because there was no operative view and it was difficult to distinguish T4 from T3 in gastric cancer by CT and PET, this slight difference was disregarded.

The NCG + CTx group displayed a higher proportion of peritoneal metastasis than the CTx group (60.0% vs. 25.0%). Distant LN metastasis was more common in the CTx group (31.6% vs. 8.0%). If we regarded the existence of mixed-type metastasis as being indicative of the aggressiveness of M1 gastric cancer, there was no significant difference in the aggressiveness of cancer between the two groups, because the mixed type metastasis between the two groups displayed no significant difference (23.7% vs. 12%, *P* = 0.266).

**Figure 3:**
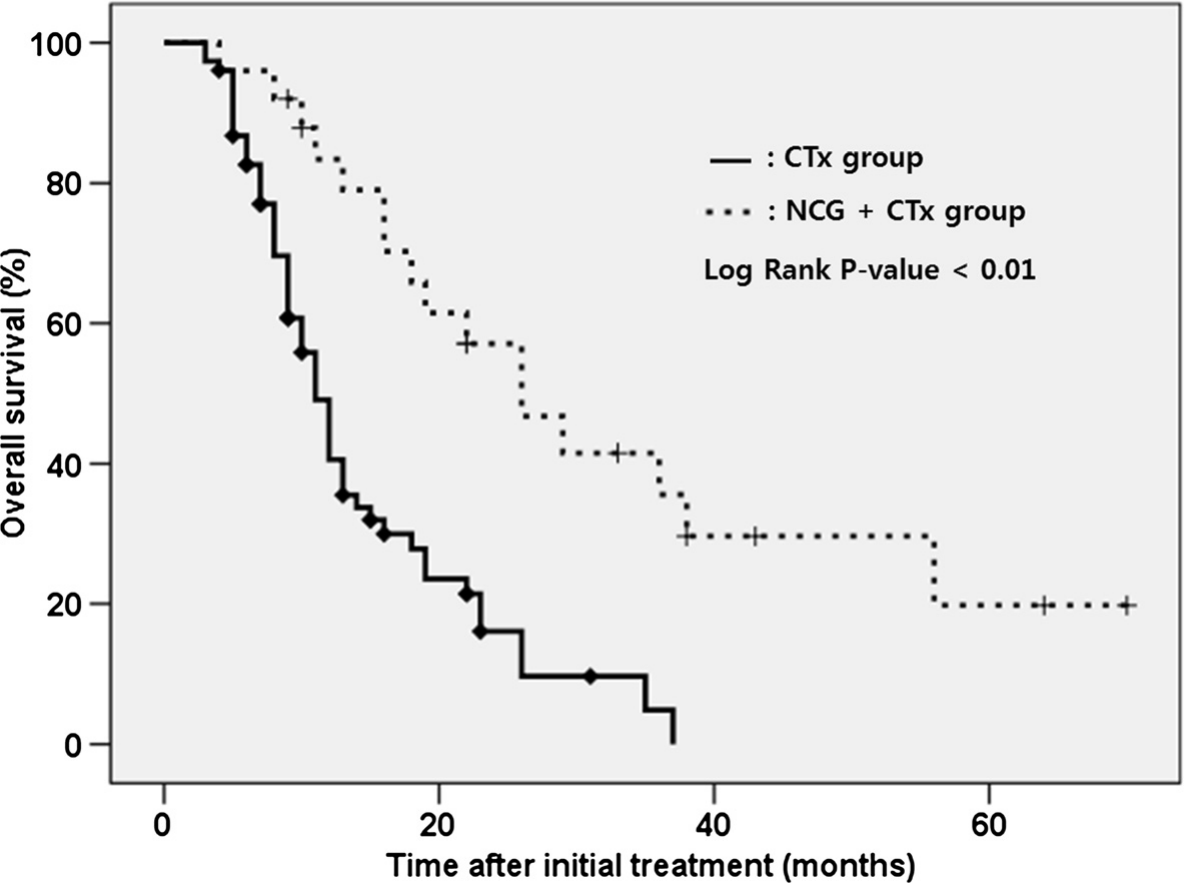
**Comparison of overall survival between the NCG + CTx group and the CTx group.**

Based on this result, we compared the chemoresponse and overall survival. The NCG + CTx group showed more favorable chemoresponse than the CTx group at both checks (60% and 72% vs. 18.4% and 23.7%; Table 3). The NCG + CTx group had a longer overall survival than the CTx group (26 vs. 11 months; Table 4, Figure 3).

**Table 3:** **Chemoresponse according to the change of regimens for chemotherapy**

**Change**	**Response**	**CTx (** ***n*** **= 76)**	**NCG + CTx (** ***n*** **= 25)**	***P*** **value**
For the first regimen	Favorable	14 (18.4%)	15 (60.0%)	<0.01
	Unfavorable	62 (81.6%)	10 (40.0%)	
Until the third regimen	Favorable	18 (23.7%)	18 (72.0%)	<0.01
	Unfavorable	58 (76.3%)	7 (28.0%)

**Table 4:** **Overall survival**

**Group**	**Median (months)**	**1-YSR (%)**	**2-YSR (%)**	**3-YSR (%)**	***P*** **value**
CTx	11	40.6	16.1	4.8	<0.01
NCG + CTx	26	83.4	57.1	35.6	

*Abbreviation*: *YSR* year survival rate.

## Discussion

Presently, patients who received chemotherapy after non-curative gastrectomy for M1 gastric cancer displayed a more favorable chemoresponse and longer overall survival. This result is consistent with the view that non-curative gastrectomy in M1 gastric cancer improves the chemoresponse by reducing the tumor burden [25,26], with minimal inhibited chemoresponse due to destruction of the anatomic route to the tumor during surgery, which lead to prolonged overall survival.

Several previous studies reported that various non-curative surgeries that reduce the tumor burden could produce a survival benefit in M1 gastric cancer [24,27-29]. However, the studies addressed survival after non-curative surgery and not chemoresponse. The significance of the present study was the focus on the relationship between non-curative gastrectomy and chemoresponse.

Generally, it is anticipated that a more favorable chemoresponse after chemotherapy could lead to longer survival. However, how the chemoresponse could influence patient survival in M1 gastric cancer is unclear [30]. This unreliable probability between chemoresponse and survival in M1 gastric cancer might be caused by the lack of an outstanding chemotherapeutic agent. With the development of such an agent, studies demonstrating the oncologic benefit of non-curative gastrectomy would be an important cornerstone for the treatment guideline for M1 gastric cancer.

This study has some limitations. The study was retrospective and the CTx group had no operative view, which could create selection bias and heterogeneity between the two groups. Second, non-curative gastrectomy in itself reduces the tumor burden, which could have overestimated a favorable response. Third, the CTx group displayed marginally more prevalent clinical T4 gastric cancer, which could be connected to poorer survival. This lack of homogeneity should be considered in overall survival comparative analysis between the two groups. Finally, a quality-of-life evaluation was not performed. Thus, it is impossible to definitely recommend non-curative gastrectomy, although it is feasible technically.

## Conclusions

Despite the aforementioned limitations due to the heterogeneity between the two groups, non-curative gastrectomy in M1 gastric cancer could improve chemoresponse and extend survival.

## Abbreviations

BMI: Body mass index
CR: Complete response
CT: Computed tomography
CTx: Chemotherapy
cT stage: Clinical T stage
5-FU: 5-Fluorouracil
LN: Lymph node
MMC: Mitomycin-C
MRI: Magnetic resonance imaging
NCG + CTx: Chemotherapy after non-curative gastrectomy
NCCN: National comprehensive cancer network
PD: Progressive disease
PET: Positron emission tomography
PR: Partial response
RECIST: Response evaluation criteria in solid tumors
SD: Stable disease
SD: Standard deviation
TS-1: Tegafur-gimeracil-oteracil potassium
YSR: Year survival rate
